# Association between *Toxoplasma gondii* Infection in Brain and a History of Depression in Suicide Decedents: A Cross-Sectional Study

**DOI:** 10.3390/pathogens10101313

**Published:** 2021-10-13

**Authors:** Cosme Alvarado-Esquivel, Laura Alejandra Mendoza-Larios, Fernando García-Dolores, Luis Francisco Sánchez-Anguiano, Elizabeth Irasema Antuna-Salcido, Jesús Hernández-Tinoco, Adriana Rocha-Salais, Marcela Araceli Segoviano-Mendoza, Antonio Sifuentes-Álvarez

**Affiliations:** 1 Biomedical Research Laboratory, Faculty of Medicine and Nutrition, Juárez University of Durango State, Durango 34000, Mexico; lfsanguiano@hotmail.com (L.F.S.-A.); jhtinoco@yahoo.com (J.H.-T.); adriana@rocha.mx (A.R.-S.); marcela_segoviano@hotmail.com (M.A.S.-M.); sifual55@hotmail.com (A.S.-Á.); 2 Amphitheater and Department of Education, Institute of Forensic Sciences, Mexico 06720, Mexico; lauraa.mendoza@tsjcdmx.gob.mx (L.A.M.-L.); fernando.garcia@tsjcdmx.gob.mx (F.G.-D.); 3 Institute for Scientific Research “Dr. Roberto Rivera Damm”, Juárez University of Durango State, Durango 34000, Mexico; petitxema@hotmail.com

**Keywords:** *Toxoplasma gondii*, suicide, cross-sectional study, immunohistochemistry, epidemiology

## Abstract

We assessed the association between *Toxoplasma gondii* (*T. gondii*) infection of the central nervous system and suicide correlates in suicide decedents. Eighty-seven decedents who died by suicide received in a forensic setting for medico-legal autopsies in Mexico City were studied. Two samples of brain (amygdala and prefrontal cortex) from each decedent were examined for detection of *T. gondii* using immunohistochemistry. Correlates of suicide including a history of previous suicide attempts, co-morbid mental disorder, consumption of alcohol or tobacco, irritability and aggression, economic problems, presence of drugs or alcohol in blood and suicide method were obtained and analyzed for their association with *T. gondii* infection. *T. gondii* immunohistochemistry was positive in prefrontal cortex sections in 6 decedents and in an amygdala section in one decedent. Thus, the prevalence of *T. gondii* infection in brain in suicide victims was 8.0% (7/87). Bivariate and logistic regression analysis of suicide correlates showed that only a history of depression was associated with *T. gondii* infection of the brain in suicide victims (OR: 12.00; 95% CI: 2.26–63.46; *p* = 0.003). Our results provide evidence that *T. gondii* infection in brain is associated with a history of depression in suicide decedents.

## 1. Introduction

Toxoplasmosis, the disease caused by the intracellular parasite *Toxoplasma gondii* (*T. gondii*), is an infection with worldwide distribution [1]. Toxoplasmosis is one of the neglected parasitic diseases in humans and animals [2]. *T. gondii* infects approximately one billion people around the world [3]. Cats and other Felidae serve as the definite host of *T. gondii* producing oocysts, whereas humans and other warm-blooded animals can serve as the intermediate host in which tissue cysts develop [4]. *T. gondii* is able to infect via ingestion of infective stages, either contained in tissue cysts or oocysts released into the environment [5]. The parasite can remarkably infect, survive and replicate in nearly all mammalian cells [6]. Infection with *T. gondii* is maintained for life in immunocompetent individuals [4]. Most human infections with *T. gondii* are mild or asymptomatic, but a life-threatening disease can occur in immunocompromised patients [6]. Some patients with primary infections with *T. gondii* may present cervical lymphadenopathy or ocular disease [7]. In addition, primary infection with *T. gondii* during pregnancy may lead to congenital toxoplasmosis with serious consequences to the fetus [8]. *T. gondii* invades and chronically persists in the central nervous system of the infected host [9]. Asymptomatic infections with *T. gondii* may have effects on behavior and other physiological processes [4]. A number of studies have shown a higher prevalence of *T. gondii* infection in individuals with various psychiatric and behavioral disorders [10]. Seroprevalence of *T. gondii* infection has been associated with aggression and impulsivity [11], obsessive-compulsive disorder [12], mixed anxiety and depressive disorder [13], schizophrenia [14,15], generalized anxiety disorder [16] and depression [17]. In addition, suicide attempts have been associated with seroprevalence of *T. gondii* infection [18] and high anti-*T. gondii* IgG antibody levels [19]. In a study of women of 20 European countries, the rates of infection with *T. gondii* were positively associated with suicide rates [20]. Extraordinarily little is known about the link between completed suicide and the presence of *T. gondii* in the central nervous system of infected hosts. Therefore, in this survey we assessed the association between *T. gondii* infection of the central nervous system and suicide correlates in a sample of decedents who committed suicide in Mexico City.

## 2. Results

In total, 87 suicide victims were included in the study of whom 20 were females and 67 were males. Their mean age was 34.8 ± 17.4 years (range: 10 to 90 years). *T. gondii* immunohistochemistry was positive in prefrontal cortex sections in six decedents and in an amygdala section in one decedent. Thus, the prevalence of *T. gondii* infection in brain in suicide victims was 8.0% (7/87). Table 1 shows results of the bivariate analysis of the characteristics of suicide victims and the prevalence of infection with *T. gondii* in brain by immunohistochemistry.

Bivariate analysis showed four characteristics of decedents with *p* values ≤ 0.20: age groups, a history of depression, schizophrenia and suicide method. These four variables were further analyzed by logistic regression analysis. This analysis showed that only a history of depression was associated with *T. gondii* infection of the brain in suicide victims (OR: 12.00; 95% CI: 2.26–63.46; *p* = 0.003). The Hosmer–Lemeshow test showed an acceptable fit of our stepwise regression analysis model (*p* = 0.63).

## 3. Discussion

Whether *T. gondii* infection is associated or not with suicidal behavior is still a matter of controversy. A positive association between *T. gondii* exposure and suicide attempts has been found in several population groups including, for instance, psychiatric patients in Korea [18] and schizophrenic patients younger than 38 years in Germany [21]. In contrast, *T. gondii* exposure was not associated with suicide attempts in adolescents in Turkey [22] and Korea [23]. Even a negative association between *T. gondii* exposure and suicidal behavior has been reported. In a study of psychiatric patients suffering from mental and behavioral disorders due to psychoactive substance use a negative association between *T. gondii* exposure and suicidal ideation was found [24]. In another study, *T. gondii* exposure was negatively associated with suicide attempts in male schizophrenic patients [25]. However, the above-mentioned studies were based on the presence of anti-*T. gondii* antibodies in serum of live persons and it is unclear whether *T. gondii* was disseminated to brain in the individuals studied and whether infection with *T. gondii* in brain was linked to suicide correlates. Therefore, in the present study we examined suicide decedents to determine the association between the presence of *T. gondii* infection in brain and suicide correlates. Of 13 variables studied, bivariate analysis showed four (age, depression, schizophrenia and suicide method) likely associated with *T. gondii* infection in brain. Analysis of these four correlates by logistic regression analysis with the backward elimination method revealed that only a history of depression was associated with *T. gondii* infection of the brain in suicide victims. We are not aware of a previous report of the association between infection with *T. gondii* in brain and depression in suicide decedents. A number of studies about the link between depression and *T. gondii* exposure in live persons have been reported and conflicting results have been found. In a case-control study of psychiatric patients in Mexico, depressed individuals had a significantly higher seroprevalence of *T. gondii* infection than controls [17]. In another case-control study, researchers found that participants who were seropositive to *T. gondii* had a significantly higher odds of being depressed compared with seronegative participants; in addition, seropositive depressed participants were more likely to have a prior history of suicide attempts compared with seronegative participants [26]. In a further study of depressed patients, investigators found that exposure to *T. gondii* was associated with increased risk of suicide attempts [27]. These findings are in line with our results. In contrast, in a meta-analysis of 29 seroepidemiological studies, researchers found that toxoplasmosis was not a risk factor for major depressive disorder [28]. A history of depression was recorded in 12 of the 87 decedents studied. It is not clear whether a history of depression was associated with suicide independently of *T. gondii* infection. It would be of interest to determine the magnitude of this association. Further research with a case-control study design to determine the association between a history of depression and suicide behavior with and without the influence of *T. gondii* infection should be conducted. A previous study of psychiatric patients in Mexico showed an association between *T. gondii* infection and depression and stratification by gender did not show a difference between males and females [17]. In the present study, we used immunohistochemistry to directly detect *T. gondii* in brain and this strategy might be better than the serology strategy used in other studies because we can get evidence of the presence of *T. gondii* in brain, whereas serology studies can only detect anti-*T. gondii* antibodies, but it is unclear whether the parasite is present in brain. In a recent case-control study conducted in Mexico City by our research team on the association between completed suicide and *T. gondii* seropositivity, we observed that the seroprevalence of *T. gondii* infection in suicide decedents was similar to the one found in decedents who died by causes other than suicide [29]. Suicide decedents included in the case-control seroprevalence study were mostly the same as those included in the present cross-sectional study. However, in the current study, our new strategy was to focus the diagnosis of *T. gondii* infection in brain by immunohistochemistry in suicide decedents. Studying brain tissues is a unique opportunity in autopsy cases and might reveal new information that serology studies cannot reveal. Results of the present study using immunohistochemistry show that *T. gondii* infection in amygdala or in prefrontal cortex is associated with completed suicide in decedents with a history of depression. We examined these brain areas because they are linked to depression [30] and prefrontal cortex is an area where *T. gondii* has been demonstrated [31]. However, *T. gondii* can be present in any area of the brain and we cannot rule out false negative results since *T. gondii* cloud be present in brain areas other than amygdala and prefrontal cortex. In addition, depression may be caused by the interaction of multiple brain regions and significant brain region alterations in major depressive disorder patients have been found in frontal lobe, thalamus, hippocampus, striatum, temporal lobe and amygdala [30]. There is scanty information on the prevalence of *T. gondii* infection in brain in Mexico. In a hospital-based autopsy series in Durango, Mexico, we found a 9.8% prevalence of *T. gondii* infection in brain by immunohistochemistry [31]. The prevalence of *T. gondii* infection in brain found in the autopsy series is comparable with the one (8.0%) found in suicide decedents in the present study.

## 4. Materials and Methods

### 4.1. Study Design, Study Population and Setting 

In this cross-sectional study, decedents who died by suicide received for medico-legal routine autopsies at the “Institute of Forensic Sciences” in Mexico City, Mexico were studied. Sampling and data collection were performed from November 2015 to December 2016. The inclusion criteria were: (1) suicide victims; (2) any age; and (3) any gender.

### 4.2. Suicide Correlates

Correlates of suicide including a history of previous suicide attempts, co-morbid mental disorder, consumption of alcohol or tobacco, irritability and aggression, economic problems, presence of drugs or alcohol in blood and suicide method were recorded. 

Information about the suicide correlates was provided by family members of suicide victims at the Public Prosecutor’s Office. In addition, other sources of information included friends and acquaintances of decedents and police. All data declared by family members and others was recorded in a file and submitted to the forensic pathologists. Data used for the present study was obtained from the files received at the forensic setting. Diagnosis of depression was based on medical diagnosis and use of medicaments against depression according to information declared by informants. 

### 4.3. Immunohistochemistry

Two brain tissues (prefrontal cortex and amygdala) from each suicide victim were collected for immunohistochemistry. Brain samples underwent formalin fixation and paraffin-embedding and were stored until analyzed. Paraffin-embedded 2 µm tissue sections were prepared for detection of *T. gondii* by immunostaining using the Tinto Detector Immuno DNA System equipment (Bio SB, Santa Barbara, CA, USA). Heat induced antigen retrieval was performed using the Digital Pressure Cooker, Model PC-2000 (Bio SB). The primary antibody “*Toxoplasma gondii*, rabbit polyclonal” (Bio SB) and the positive control “*Toxoplasma gondii* positive control slides” (Bio SB) were used. The Mouse/Rabbit ImmunoDetector HRP/DAB (Bio SB) was used with the rabbit primary antibody. Immunohistochemistry staining was performed as per manufacturer’s instructions. Immunohistochemistry slides were evaluated by a pathologist (LFSA).

### 4.4. Statistical Analysis

Data were analyzed with the aid of the software IBM SPSS Statistics version 20.0 (IBM Corp, Armonk, NY, USA) and Epi Info 7 (Centers for Disease Control and Prevention, USA). A sample size of 87 was calculated based on a population size of about 240 suicide decedents received at the participating forensic setting a year, an expected frequency of 9.8% of *T. gondii* infection [31], confidence limits of 5%, a design effect of 1.0, one cluster and a confidence level of 95%. We used the Pearson’s chi-square test and the Fisher exact test (when values were 5 or less) to determine the association between results of immunohistochemistry and suicide correlates. Variables with *p* values ≤ 0.20 obtained in the bivariate analysis were further analyzed using logistic regression analysis with the backward stepwise method. Independent variables included in the regression analysis model were age groups, depression, schizophrenia and suicide methods. Goodness of fit of our logistic regression model was evaluated with the Hosmer–Lemeshow test. We calculated odds ratios (OR) and 95% confidence intervals (CI) and a *p* < 0.05 was considered as statistically significant. 

## 5. Conclusions

Our results provide evidence that *T. gondii* infection in brain is associated with a history of depression in suicide decedents. Further research to confirm the association between *T. gondii* infection in brain, depression and completed suicide should be conducted.

## Figures and Tables

**Table 1:** Bivariate analysis of infection with *T. gondii* in brain and characteristics of decedents who committed suicide.

Characteristic	Decedents Tested No.	Prevalence of *T. gondii* Infection		*p* Value
		No.	%	
Sex				
Male	67	5	7.5	0.65
Female	20	2	10.0	
Age groups (years)				
30 or less	42	1	2.4	0.08
31–50	30	5	16.7	
51 or more	15	1	6.7	
Previous suicide attempts				
Yes	5	1	20.0	0.35
No	81	6	7.4	
Depression				
Yes	12	4	33.3	0.006
No	75	3	4.0	
Schizophrenia				
Yes	2	1	50.0	0.15
No	85	6	7.1	
Anxiety				
Yes	2	0	0.0	1.00
No	85	7	8.2	
Alcohol consumption				
Yes	41	3	7.3	1.00
No	40	3	7.5	
Tobacco consumption				
Yes	35	2	5.7	0.45
No	45	5	11.1	
Alcohol in blood				
Yes	28	2	7.1	1.00
No	54	4	7.4	
Controlled substance in blood				
Yes	32	2	6.2	1.00
No	50	4	8.0	
Irritability and aggression				
Yes	10	1	10.0	0.58
No	77	6	7.8	
Economic problems				
Yes	4	1	25.0	0.28
No	83	6	7.2	
Method of suicide				
Bleeding	3	1	33.3	0.19
Firearm	5	0	0.0	
Hanging	73	5	6.8	
Injuries	3	1	33.3	
Poisoning	3	0	0.0	

## Data Availability

Data are provided within the article.
